# *P*-Value, Confidence Intervals, and Statistical Inference: A New Dataset of Misinterpretation

**DOI:** 10.3389/fpsyg.2018.00868

**Published:** 2018-06-08

**Authors:** Ziyang Lyu, Kaiping Peng, Chuan-Peng Hu

**Affiliations:** ^1^Department of Psychology, School of Social Science, Tsinghua University, Beijing, China; ^2^Neuroimaging Center (NIC), Focus Program Translational Neuroscience (FTN), Johannes Gutenberg University, Mainz, Germany; ^3^Deutsches Resilienz Zentrum (DRZ), University Medical Center of the Johannes Gutenberg University, Mainz, Germany

**Keywords:** *P*-Value, confidence intervals (CIs), misinterpretation, replication crisis, statistical inference

## Introduction

Statistical inference is essential for science since the twentieth century (Salsburg, 2001). Since it's introduction into science, the null hypothesis significance testing (NHST), in which the *P*-value serves as the index of “statistically significant,” is the most widely used statistical method in psychology (Sterling et al., 1995; Cumming et al., 2007), as well as other fields (Wasserstein and Lazar, 2016). However, surveys consistently showed that researchers in psychology may not able to interpret *P*-value and related statistical procedures correctly (Oakes, 1986; Haller and Krauss, 2002; Hoekstra et al., 2014; Badenes-Ribera et al., 2016). Even worse, these misinterpretations of *P*-value may cause the abuse of *P*-value, for example, *P*-hacking (Simmons et al., 2011; John et al., 2012).

To counter these misinterpretations and abuse of *P*-values, researchers have proposed many solutions. For example, complementing NHST with estimation-based statistics (Wilkinson and the Task Force on Statistical Inference, 1999; Cumming, 2014), lower the threshold for “significance” (Benjamin et al., 2017) or totally banning the use of NHST and related procedures (Trafimow and Marks, 2015) and using Bayes Factor (Wagenmakers et al., 2011, 2017). Of all these solutions, the estimation-based statistics was adopted by several mainstream psychological journals. One reason is that confidence intervals (CIs) of the estimation-based statistics help better statistical inference (though not guarantee it) (Coulson et al., 2010). However, the first step of changing is to know to what extent people in the field misinterpreting these statistical indices and how the misinterpretations caused abuse of these statistical procedures in research.

Here we introduce the raw data available for anyone who is interested in examining how students and researchers misinterpret of *P*-value and CIs, as well as how NHST and CIs influence the interpretation of research results. Part of the results had been reported in our previous Chinese paper (Hu et al., 2016).

## Materials and methods

### Participants

Participants (*N* = 362, 208 females, 153 males, 1 unanswered, age: 25.65 ± 6.65 years) took part in the survey through online surveys or a paper-pen survey.

As for the online survey, we recruited participants through social media (include WeChat, Weibo, blogs etc.), without any monetary or other material payment. Data were collected at different periods: the 1st online dataset was collected from August 2015 to October 2015; the 2nd online dataset was collected in October 2015; the 3rd online dataset was collected from December 2016 to January 2017. Given that our focus is on psychological researchers, we asked participants to indicate whether or not they were from psychology or related fields (such as cognitive neuroscience, psychiatry or educational science). If they indicated that they were not from psychology or related fields, the survey then ended automatically (first two on-line surveys), or they continued without interruption but their data were excluded from the valid dataset (the third on-line survey). In total, data from 246 participants were included in valid dataset from the online surveys, while 146 participants were either prevented from answering the questions (128 participants) or excluded from the valid dataset (18 participants).

The paper-pen survey data were collected during the registration day of the 18th National Academic Congress of Psychology, Tianjin, China, in October 2015. Attendees of the conference were invited to participate the survey after they finished the conference registration. If attendees agreed, they were invited to fill a single-page questionnaire immediately. Data from participants who didn't finish all questions concerning *P-*value or CIs were excluded. The detailed information of our sample, please see Table 1.

**Table 1:** Information of respondents.

**Education**	**Gender**	*P-*value^*^	**CIs^†^**	**Inference^‡^**
Undergraduate	Female	73	73	69
	Male	34	34	34
Master or Junior PhD student	Female	95	95	34
	Male	62	62	38
Senior PhD student	Female	28	28	21
	Male	33	33	26
With PhD Degree	Female	12	12	8
	Male	24	24	16

* P-value = Number of respondents answered the questions about interpretations of P-value;

† = Number of respondents answered the questions about interpretations of CIs;

‡ *= Number of respondents answered the questions about inference based on results of two studies*.

This study was approved by the local ethics committee (Ethical Committee of the Department of Psychology, Tsinghua University, Beijing, China). A written informed consent in accordance with the Declaration of Helsinki were presented (online survey) or read by an experimenter (for paper-pen survey) to respondents before they began the survey.

## Materials

The materials included three parts, all of them were translated by C-P Hu and reviewed by colleagues.

### The interpretation of *P*-value

In this question, respondents first read a fictitious research scenario, where the *P*-value is equal to 0.01. Then they were presented with six statements about *P*-value and were required to “mark each of the statements as “true” or “false.” “False” means that the statement does not follow logically from the above premises.” This question was translated from previous studies (Haller and Krauss, 2002; Gigerenzer, 2004). (Note: we corrected the degree of freedom from 18 to 38, because 38 is the right one for two-sample *t*-test with two independent 20-subject groups). All six statements were “false,” therefore participant's response was regarded as wrong if it indicated any of the statements as “true.” The percentage of participants who made at least one error as the overall error rate on this question.

### The interpretation of confidence intervals (CIs)

Similar to the above one, this question describes a fictitious scenario in which a researcher conducts an experiment and reports a 95% confidence interval for the mean that ranges from 0.1 to 0.4. As in Hoekstra et al. (2014), either the topic of the study or the underlying statistical model in the fictitious experiment was specified. After reading the scenario, respondents were asked whether or not they endorse six statements that representing possible misconceptions of the CI. Similar to the *P*-value question, the “False” was also defined as a statement that does not follow logically from the scenario's result. Therefore, the correct proportion of endorse item should be zero. This question was translated from Hoekstra et al. (2014).

### How NHST and CIs influence the inference based on results of two studies

This question, which was translated from Coulson et al. (2010), investigated the effect of NHST or CIs on the interpretation of two experiments. In this fictitious scenario, two experiments were conducted to compare a new treatment for insomnia with current treatment, one experiment showed that mean difference was 3.61, *p* = 0.02, 95% CI [0.61 6.61], while the other experiment found mean difference of 2.23, *p* = 0.22, 95% CI [−1.41 5.87]. As in Coulson et al. (2010), these results were shown in four different formats: CI figure, CI text, NHST figure, or NHST text.

Participants were randomly assigned to one of four conditions, and were asked to rate their attitude on a scale from 1 (strongly disagree) to 7 (strongly agree) to following statements: “the results of the two studies are broadly consistent” (mentioned as “broadly consistent” below); “there is reasonable evidence the new treatment is more effective” (mentioned as “more effective” below); “there is conflicting evidence about the effectiveness of the new treatment” (mentioned as “conflict” below).

There are 65 participants (31 female, age: 25.15 ± 5.27 years) for the CI figure condition; 65 participants (36 female, age: 24.89 ± 4.76 years) for the CI text condition; 81 participants (48 female, age: 25.18 ± 5.14 years) for NHST figure condition; 35 participants (17 female, age: 25.11 ± 4.90 years) for the NHST text condition.

## Descriptive results

**Figure 1:**
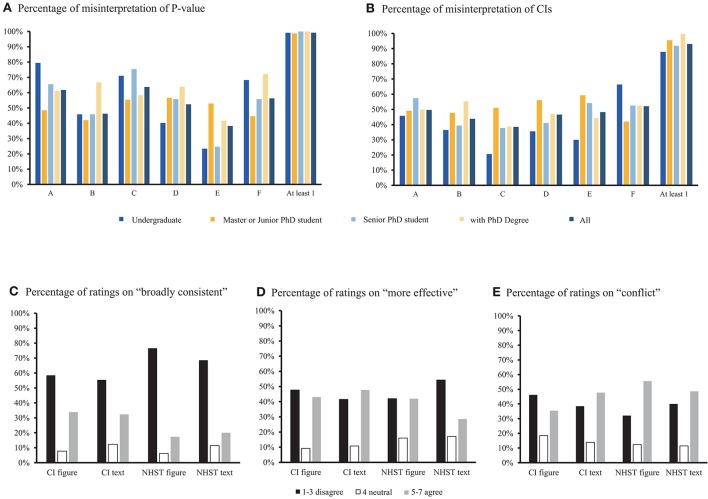
Descriptive results from all valid data. **(A)** Percentage of respondents who misinterpreted the *P*-value, the “A” to “F” on X-axis represent six statements about *P*-value, “At least 1” means that respondents misinterpreted at least one statement; **(B)** Percentage of respondents who misinterpreted the CIs, the “A” to “F” on X-axis represent six statements about *P*-value, “At least 1” means that respondents misinterpreted at least one statement; **(C)** Response for the question on “broadly consistent” (in percentage); **(D)** Response for the question on “more effective”(in percentage); **(E)** Response for the question on “conflict” (in percentage).

The data indicates that 99% subjects have at least 1 wrong answer of *P*-value understanding (Figure 1A) and 93% subjects have at least 1 wrong answer of CI understanding (Figure 1B).

For the statement that the two studies are “broadly consistent,” more respondents agreed when the result presented in CI (CI figure, 34%, CI text, 32%) than in NHST (NHST figure 17%, NHST text 20%; see Figure 1C). For the statement that the new treatment is “more effective,” more respondents agreed when results presented in CI (CI figure, 43%, CI with text, 48%) than in NHST (NHST figure 42%, NHST text 29%; see Figure 1D); For the statement that the two studies are “conflict,” there were more respondent agreed when results presented in NHST (NHST figure 56%, NHST text 49%) than in CI (CI figure, 35%, CI text, 48%; see Figure 1E).

## Reuse potential

Improving the ability to making statistical inferences is crucial for next-generation psychologists. Knowing how students and young researchers understand the most-used statistic technique is the first step to make the improvements happen. Therefore, this dataset can be used for educational purposes, e.g., as an illustration of how psychology students and researchers interpret *P*-value and CIs wordwild. Or, it can serve as a baseline for further studies, to compare whether or not the statistical inference ability improved over time (e.g., through the online course *Improving your statistical inferences*: https://www.coursera.org/learn/statistical-inferences). Moreover, it can serve as the descriptive data of students and researchers in psychology when doing cross-fields comparisons (Greenland et al., 2016).

Another potential reuse of the current dataset is to explore the relationship between the interpretation of *P*-value/CIs (question 1 and 2 of the dataset) and inference based on results from two studies (question 3 of the dataset). A previous study showed that CIs can help but not guarantee respondents better evaluation of the results from two studies (Coulson et al., 2010), but it is unknown that whether the effect of CIs on statistical inference was due to a better understanding of CIs, or due misunderstanding of CIs (Morey et al., 2016). The current dataset provides information on both understanding of NHST (question 1)/CIs (question 2) and statistical inference (question 3), therefore can serve as pilot data for further studies.

It is worth mentioning that the reliability of the questions in the current dataset is low^1^. This is not surprising, however, as these items were developed without considering their psychometrical properties in previous studies. Also, there are only six items for each survey and each item was designed to test a different aspect of the understanding of *P*-value or CI, thus, the homogeneity of the questions are low. Our data could be used in future studies that aimed at developing a psychometrically valid and reliable survey to measure how accurately the statistical inferences researchers can make.

## Author contributions

C-PH conceived the idea. C-PH collected the data. ZL and C-PH preprocessed the data and uploaded data to osf.io. ZL, KP, and C-PH prepared the manuscript. All authors reviewed and approved the manuscript.

## Supplementary

The raw data are deposited at https://osf.io/vnjye/ in excel format, including four data files and two codebooks (Online survey data named “codebook_online_data.xlsx”, “Online_data_3_201612_EN_de_ID.xlsx”, “Online_data_2_201510 _EN_de_ID.xlsx”, and “Online_data_1_201510_EN_de_ID.xlsx”; Paper survey data named “codebook_paper_data.xlsx” and “Online_data_3_201612_EN_de_ID.xlsx.”).

We also deposited the cleaned data and the corresponding codebook: (“Valid_data_Do_you_real_understand_p-value_and _CIs_EN_201710.xlsx” & “codebook_valid_data.xlsx”), which include all the valid responds.

### Conflict of interest statement

The authors declare that the research was conducted in the absence of any commercial or financial relationships that could be construed as a potential conflict of interest.

## Footnotes

^1^We analyzed the α coefficient of both the questionnaires about p-value and CI, and found that the overall α coefficient are −0.007 and 0.452, respectively.
